# An Address to the Graduates of the Medical Department of the St. Louis University

**Published:** 1851-05

**Authors:** 


ART. XII.—*An Address* to the Graduates of the Medical Department of the St. Louis University. By A. LITTON, M. D., Professor of Chemistry and Pharmacy.

*Valedictory Address* before the Graduates of Washington University, Baltimore. By Professor T. E. BOND.

*Valedictory Address* to the Graduates of the Medical Department of the University of Pennsylvania. By W. E. HORNER, M. D., Professor of Anatomy.

*The prospective progress of Medicine in America:* a valedictory address delivered before the graduating class at the first annual commencement of the N. Y. Medical Colleges. By Horace Green, M. D., Professor of Theory and Practice of Medicine.

We have grouped these various addresses together, and take much pleasure in bearing testimony to their merits The following picture of a medical practitioner, from Dr. Litton's address, will be recognised by many as a very faithful one:

The physician is the servant of the high and the low, the rich and the poor; and must be obedient to their call, however unreasonable and capricious. Night, that to others brings the rest and the quietness that nature's tired powers demand, that to beast and to bird is the time of unwearied repose, gives to him no assurance that the slumber of sleep shall be undisturbed. Disease stays not its march to consult his desires or his pleasures; but calls him from the hall of festivity, from the amenities of the social circle and from the holy temple of religious worship. The storm and the tempest, the piercing cold and the bitter wind, that drives labor from its toil to nestle around its warm fire-side, and force the beast to seek his lair and the bird its nest, delay not his visit; but go he must, oftimes for long and weary miles, through cheerless and deserted tracts, that he may cool the burning brow, or bind up the broken limb of his fellow man. Pestilence, that causes the stout heart to quail and that makes friendship, strengthened by the mutual charities of life and cemented by years of devotion, falter, and, in this hour of need, deny its brother; however terrible its ravages, and desolating its sway, cannot stop him; but he must go to infected hospitals, to dwellings crowded with the victims of the destroying angel, to places polluted with the dead and the dying, unconscious at what moment the archer's invisible shaft may pierce his heart, and force him to leave his wife a widow, and his children orphans, to continue unsupported and unsustained, their dreary pilgrimage of life. Others, with the power of steam, and the speed of railroad, and the wings of the wind, may seek places and climes not infected; but the physician stands a sentinel upon the watch-tower, to guard the citadel of life, and cannot, dare not, and, God trust, would not be so meanly base, as recreantly to desert those whom hard fate chains to infected regions, and leave them unprotected and unguarded to the fell destroyer's rage."

The following parallel between the law and medical professions, drawn by Dr. Bond is done with truth and spirit:

When the advocate for a client, indicted for a capital crime, stands up to speak in his defence, he must be less than human if he does not tremble under the weight of his undertaking. But serious as is his responsibility, it is shared by others. A mistake might not be mischievous; the Court might correct his law, and the hearts of the jury add force to his pleadings. But in *his* fearful office the physician stands alone. Hastily summoned—perhaps at the dead of night—without a moment's preparation, with no intelligible information as to the nature and extent of the demand to be made upon his faculties, he is hurried to the chamber where death has begun his work. However terrible, however complicated, however unusual the disease, the physician must meet the exigency, and meet it promptly. * * * After you shall have saved a life from the greatest peril, after you shall have restored a citizen to the State, a father to his children, a husband to his wife, you will have done precisely what you were expected to do—what you were sent for to do, and no more will be thought of the matter. In a little while it is quite likely that you will find yourself superseded by another physician, every way your inferior. But he may have flattered the family by some insincere attentions, or astonished them by a parade of learning—he may have been heard to call the back-bone the spine, or the brain the cerebrum, or a bruise an ecchymosis—or he may have won their confidence by the narration of some wonderful cures—or he may be a new man who has lately joined the same church and claims the practice of the brotherhood upon ecclesiastical principles—being determined not to serve God for naught. Or you may be discarded to make room for a mere mountebank, who may have made himself a doctor by the force of his will, and business by the force of his impudence—an utter ignoramus, with no more knowledge of disease than of the means of curing it, and no more of either than has the more respectable animal that drags him through his daily rounds of mortal visitation.

The address of Professor Horner completed his fortieth year as a medical lecturer, and his address is replete with sound advice, accurate observation, and well matured wisdom. Long may he continue to adorn the station he has long filled with credit to himself, and usefulness to thousands.

Dr. Green did not do his subject ample justice, in his omission of all reference to what the West and South have done for the progress of medicine in America. If he had been familiar with the history of what the physicians and surgeons of those regions have done, he would have been able to throw a flood of light upon the principal features of his address. In another part of this Journal, in a notice of a statement recently made by Professor Peaslee, we have endeavored to vindicate the West, against the continued efforts of the East, to be oblivious to her merits. In addition to what is recorded in that vindication, we ask Dr. Green's attention to the following remarks, contained in a recent publication made by Prof. J. B. Flint:

From the date of the earliest surgical records to the present time, various plans have been proposed and executed for the radical cure of Hernia, by operations with the knife or cautery, by excision, scarification, organic plugs, etc. etc., all of which are acknowledged to have been ineffectual, and many of them absurd or barbarous in the extreme. Velpeau, in his great work on operative surgery, devotes fifteen ample pages to a description of these various processes, which, if their number and failure prove the disease against which they are directed, to have been among the opprobria medicinæ, abundantly convict themselves of being among the opprobriæ medicorum. When I mention that among these processes, that which had been employed more extensively than any other one, was castration—when, we refer to the astonishing submission to revolting measures which the history of this matter reveals, nothing need be added to show how strong and general has been the desire for a radical cure of this common infirmity. Modern surgery had repudiated the abominations to which I refer, but had afforded no effectual substitute, and had become content with palliative measures as the best that could be done.

The treatment of reducible Hernia as practised by the most experienced men of the profession, may be summed up in the pithy sentence of Abernethy—"You return it" —the bowel—"put a truss on it and keep it on."

Sometime between the years 1830 and '35, a Mr. Stagner of Kentucky, experimenting on his own person, arrived at the discovery which is at the foundation of the present method for the radical cure of Hernia, by means of trusses with wooden pads.

Dr. Hood, a neighboring physician of respectable standing, was not slow to appreciate the value of Stagner's discovery, and, bringing to the application of it his anatomical and surgical knowledge, constructed a truss with which he accomplished a cure in a considerable number of cases. He went to Philadelphia, and became associated with Dr. Heber Chase, whose mechanical turn of mind and accurate professional knowledge, eminently fitted him to impress upon the new instrument the modifications necessary to adapt it to the various forms of Hernia, and to the particular anatomical relations of each. An admirable set of instruments was the result of this partnership —the essential element in their structure, being the Kentucky discovery—which constitute, as I believe, the most valuable improvement in our materia chirurgica that has appeared since the introduction of the ligature as an hæmostatic instrument. All the old fashioned trusses were applied with a view only to retention, and although a cure of the disease sometimes happened under their use, this was not the purpose, nor expectation of it. Soon after Dr. Chase had completed his instruments, they were submitted to a special committee of the Philadelphia Medical Society, appointed to investigate the subject of the radical cure of Hernia. At the head of this committee was Dr. Reynold Coates, a highly philosophical and excellent surgeon, who devoted himself, and engaged his colleagues for many months in investigating the claims of the new instruments. They examined many patients who had been suffering with rupture for many years, and found a very large portion of them perfectly cured, after wearing the wooden-block truss for a certain number of weeks or months. Their report was highly favorable to the claims of the new instrument, and their conclusions have been verified by other practitioners, whenever a faithful and intelligent employment of it has been tried. From all the evidence which has been collected and published on the subject, I believe we are warranted in concluding that, of any *ten* cases of Hernia, not having any extraordinary difficulties to overcome, *eight* may very certainly be radically cured by a judicious employment of the truss, the invention of which must fairly be reckoned among the contributions of Kentucky to the surgical therapeutics of our favored era.

We seek no particular laudation of the West and South, but we demand that justice shall be meted out to their strong and brilliant claims to the respect and admiration of their professional brethren. The West and South never withold their approbation of their Eastern brethren, but take pleasure in their success and advancement, and this proper conduct should be reciprocated. B.

